# Rho GTPase signalling in cell migration

**DOI:** 10.1016/j.ceb.2015.08.005

**Published:** 2015-10

**Authors:** Anne J Ridley

**Affiliations:** Randall Division of Cell and Molecular Biophysics, King's College London, New Hunt's House, Guy's Campus, London SE1 1UL, UK

## Abstract

Cells migrate in multiple different ways depending on their environment, which includes the extracellular matrix composition, interactions with other cells, and chemical stimuli. For all types of cell migration, Rho GTPases play a central role, although the relative contribution of each Rho GTPase depends on the environment and cell type. Here, I review recent advances in our understanding of how Rho GTPases contribute to different types of migration, comparing lamellipodium-driven versus bleb-driven migration modes. I also describe how cells migrate across the endothelium. In addition to Rho, Rac and Cdc42, which are well known to regulate migration, I discuss the roles of other less-well characterized members of the Rho family.

**Current Opinion in Cell Biology** 2015, **36**:103–112

This review comes from a themed issue on **Cell adhesion and migration**

Edited by **Michael Sixt** and **Erez Raz**

For a complete overview see the Issue and the Editorial

Available online 10th September 2015

**http://dx.doi.org/10.1016/j.ceb.2015.08.005**

0955-0674/© 2015 The Author. Published by Elsevier Ltd. This is an open access article under the CC BY license (http://creativecommons.org/licenses/by/4.0/).

## Introduction

Cell migration is essential for the development of multicellular animals. During development, some cell populations migrate long distances, for example neural crest cells migrate throughout the embryo to form different kinds of cells such as melanocytes, vascular smooth muscle and Schwann cells [1]. Cell migration also contributes to progression of most human diseases. Cancer cells migrate into lymph nodes or blood vessels to form metastases [2], while immune cell migration is central to autoimmune diseases and chronic inflammation [3].

Over the last few years it has become clear that cells are highly flexible in the ways they migrate, and can change rapidly between different migration modes. Cells can migrate as single cells or collectively as groups [4]. They interchange between lamellipodium-based and bleb-based motility depending on the stiffness and composition of their environment, including extracellular matrix components and surrounding cells [5, 6]. Cell•cell interactions strongly affect how cells move and what regulates their migration. When a cell meets another cell, they often stop migrating in a process called contact inhibition, and either form cell•cell adhesions or change direction, leading to cell dispersal *in vivo* [7]. Cells may be guided towards a particular place by soluble or matrix-associated signals, or may apparently migrate randomly with frequent direction changes [8]. What is common to all these modes of migration is the involvement of Rho GTPases.

Rho GTPases were first identified to have roles in cell migration around 20 years ago [9]. Many experiments use cells migrating on 2-dimensional (2D) substrata *in vitro*, but more recent work in 3-dimensional (3D) environments *in vitro* and in animals *in vivo* have considerably expanded our understanding of how different Rho GTPases contribute to cell migration through tissues and tissue-like environments.

There are 20 Rho GTPase genes in humans (Table 1). Most Rho GTPases are active and stimulate their downstream targets when bound to GTP, and inactive when bound to GDP. They are activated by guanine nucleotide exchange factors (GEFs), which induce exchange of GDP for GTP, and inactivated by GTPase-activating proteins (GAPs), which catalyse the hydrolysis of GTP to GDP on Rho proteins. The best studied Rho GTPases, Rho, Rac and Cdc42, are the most highly conserved Rho family members across eukaryotic species, being found in plants, fungi and/or animals [10]. They contribute to cell migration in all animal model organisms tested, but continue to provide surprises on their multiple roles in cell migration. In humans, there are three closely related Rho and Rac genes, and splice variants of Rac1 and Cdc42 increase the diversity of proteins (Table 1), complicating the analysis of how each protein contributes to migration. In addition, there are 13 other Rho family members in mammals, which have diverse and much less well characterized roles in cell migration.

Here, I describe the roles of Rho family proteins in animal cell migration, using information from both *in vitro* and *in vivo* models.

## Lamellipodium-driven migration

**Figure 1:**
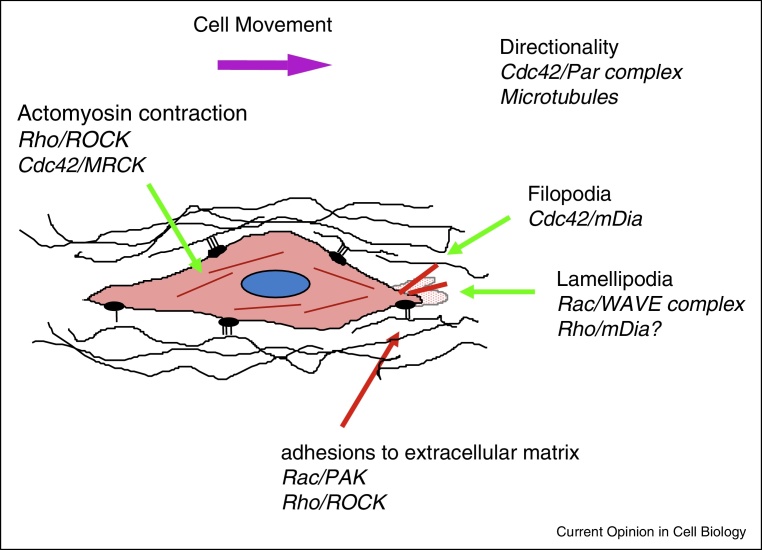
Rho GTPases in lamellipodium-driven migration. In cells using lamellipodia to drive migration, cell migratory polarity is established by Cdc42, acting through the Par polarity complex and microtubules. Membrane protrusions at the front of cells include lamellipodia and filopodia. Cdc42 is the main GTPase contributing to filopodium extension, acting through mDia formins. Rac induces lamellipodium extension through the WAVE complex, which activates the Arp2/3 complex. Adhesions to the extracellular matrix form in lamellipodia, initially through Rac and its target PAK, among other proteins. Rho and ROCKs promote formation of larger, more persistent integrin-based adhesions. Actomyosin contraction in the cell body is important for driving the cell forward and for detachment of the back of the cell, and is mediated by Rho and ROCKs and/or Cdc42 and MRCKs.

**Figure 2:**
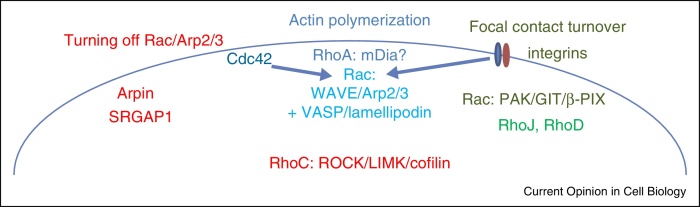
Signalling in lamellipodia. In lamellipodium-driven migration, actin polymerization at the front of cells requires Rac, which recruits the WAVE complex to activate Arp2/3 complex-mediated actin polymerization. VASP and the adaptor protein lamellipodin (which interacts with VASP, Rac and the WAVE complex) contribute to actin polymerization. RhoA is also active at the front of extending lamellipodia, and might contribute to actin polymerization through a formin such as mDia1. Cdc42 and integrins contribute to inducing and maintaining active Rac selectively at the leading edge of migrating cells. Negative feedback loops restrict the extent of Rac activation, including Arpin (which inhibits the Arp2/3 complex) and SrGAP1 (a GAP for Rac). RhoC acts further back in the cell, behind Rac, to downregulate cofilin activity (via LIMK) and hence decrease actin polymerization, and stimulate actomyosin contractility (via ROCK), which pulls the lamellipodial network rearwards. During migration, integrin-based focal contacts need to be turned over, and this involves Rac itself, acting through a PAK/GIT/β-PIX complex that is localized to focal contacts. RhoJ and RhoD also contribute to focal contact turnover.

Plasma membrane extension in lamellipodia is driven predominantly through Rac-mediated actin polymerization (Figure 1, Figure 2). In order for lamellipodia to contribute productively to cell migration, lamellipodial protrusion needs to be limited to one part of the plasma membrane. In 3D environments, slow moving cells such as fibroblasts can extend lamellipodia [11]. Lamellipodia are frequently observed at the front of single cells migrating *in vivo*, as well as at the front of leading cell(s) of collectively migrating cells. For example, dendritic cells use lamellipodia to crawl along lymphatic endothelial vessels towards lymph nodes following activation in the tissues [12], and cells at the front of collectively migrating *Drosophila* border cells extend long Rac-driven lamellipodia [13]. Integrin-mediated adhesion is generally considered essential for lamellipodium-driven migration, in part because it perpetuates Rac activation in a positive feedback loop, in which engagement of integrins at the leading edge stimulates Rac activation [14]. By contrast, in situations of low adhesion or if cells lack integrins, cells tend to migrate using bleb-based motility [5].

### Actin polymerization in lamellipodia

Under normal conditions, lamellipodium-driven migration requires active Rac proteins (Rac1, Rac2 and/or Rac3 depending on the cell type and conditions), and indeed local Rac activation is sufficient to drive migration *in vivo* [13, 15]. Several Rac GEFs are involved in activating Rac to induce lamellipodia, including Tiam1, β-PIX, and DOCK180 [16, 17]. Active Rac proteins interact with a WAVE-associated complex of proteins (Figure 1, Figure 2), which in turn activates actin nucleation by the Arp2/3 complex. Active Rac interacts with the scaffold protein lamellipodin, which contributes to actin filament extension in lamellipodia by binding to the WAVE complex [18•, 19], and hence may act to bring Rac close to the WAVE complex.

The actin polymerization in lamellipodia involves not only the Arp2/3 complex but also formins and VASP (Figure 2) [20, 21]. Rac proteins interact with several formins [20], but whether these interactions contribute to lamellipodia or cell migration is not clear. The WAVE complex can also interact directly with VASP, and this interaction is important for lamellipodium formation in *Caenorhabditis elegans* embryogenesis [22^•^]. Altogether, these results indicate a complex network of proteins acting to regulate lamellipodium extension (Figure 2).

In addition to Rac, RhoA and Cdc42 are active in lamellipodial regions and contribute to lamellipodium extension [23, 24]. RhoA is activated right at the front of lamellipodia [25]. It is thought that RhoA activates formins such as mDia proteins at the leading edge of lamellipodia (Figure 2), but this has not been proven so far.

Lamellipodia are not essential for migration, and indeed melanoblasts and fibroblasts can migrate without Rac or the Arp2/3 complex, albeit more slowly [26, 27, 28]. In the absence of Arp2/3 complex, fibroblasts predominantly use filopodia to migrate [26, 27]. Melanoblasts use short stubby protrusions, which might be driven by formins [28]. Cells lacking WAVE2 have severely impaired lamellipodium formation and reduced migration [29], and WAVE1 and WAVE3 may have different functions in regulating actin dynamics [29, 30, 31]. However, *Dictyostelium* cells can still form lamellipodia in the absence of the WAVE complex. In these cells, WASP is recruited to the leading edge by Rac and activates the Arp2/3 complex [32]. Rac is more active in the absence of WAVE complex components, possibly because a negative feedback loop involving the WAVE complex is not present.

A fine balance is needed between actin polymerization and adhesion to allow productive lamellipodium-based migration. The Rac/Cdc42-activated PAK family of kinases play key roles in promoting integrin-based adhesion turnover (Figure 1) [33]. Three relatively little-characterized Rho GTPases also contribute to this balance. RhoJ regulates endothelial cell motility by promoting endothelial focal adhesion disassembly and reducing actomyosin contractility [34, 35]. RhoJ interacts with a GIT/β-PIX complex at focal adhesions to stimulate their disassembly. β-PIX is a GEF for Rac1 and Cdc42 [16] and RhoJ and β-PIX also interact with Rac/Cdc42-activated PAK kinases [33, 36], but whether RhoJ activates either of these proteins is not known. RhoD depletion similarly increases focal adhesions and reduces cell migration [37], although again the mechanism is not known (Figure 2). RhoH is highly expressed in haematopoietic cells, which migrate very fast *in vivo*. RhoH inhibits adhesion via the T-cell integrin LFA-1 (αLβ2), which could contribute to its role in cell migration [38]. Altogether, it is clear that cells have multiple ways to regulate adhesion turnover during migration. Different Rho family members may be used depending on the cell type and other signalling inputs.

### Limiting Rac activity during lamellipodium-driven migration

As mentioned above, it is crucial to limit actin polymerization to one part of the plasma membrane for a cell to move productively using lamellipodium-driven migration. This is generally believed to involve restricting Rac activity, which can occur by several different mechanisms. First, Cdc42 plays an important role in establishing cell migratory polarity and migratory persistence, acting through the Par polarity complex (Figure 1) as well as other targets. Cdc42 can localize Rac activity through multiple potentially synergistic pathways including microtubule capture at the leading edge, RacGEF localization and directed vesicle trafficking [39]. Second, feedback loops involving Rho/ROCK and actomyosin contractility are postulated to turn off lamellipodia in other regions of the cell, and indeed reducing RhoA, RhoC or ROCK activity can lead to multiple and/or larger lamellipodia [40], indicating the importance of balancing lamellipodia with contractility in lamellipodium-driven migration. RhoC is important for acute lamellipodium extension in response to EGF, and acts behind the lamellipodium, at least in part to turn off cofilin activity away from the leading edge [41]. In this model, cofilin severs existing actin filaments to initiate Arp2/3-driven actin polymerization in lamellipodia [41]. Similarly, in migration in 3D, RhoC plays an important role in inhibiting cofilin activity around invadopodia, thereby restricting cofilin-induced generation of actin filament barbed ends to the core of invadopodia [42]. Keratinocytes lacking RhoA are defective in directed migration [43], suggesting that it regulates where Rac proteins are active. Macrophages lacking RhoA and RhoB (and do not express RhoC) apparently have no defect in lamellipodial protrusion but a defect in lamellipodial retraction and tail retraction [44^•^]. This is consistent with RhoA/B activating ROCKs and hence increasing levels of actomyosin contractility at the front and back of cells. Surprisingly, RhoA/B-null macrophages migrate faster in 2D *in vitro* and get recruited into tissues more rapidly *in vivo*. Interestingly, levels of phosphorylated MLC (a measure of actomyosin contractility) increased rather than decreased in RhoA/RhoB-null cells [44^•^], suggesting the cells could compensate for lack of Rho proteins by upregulating other signalling pathways that affect MLC phosphorylation. Whether there is also compensation for Rho function in lamellipodia through increased expression of another Rho GTPase such as RhoF, which is known to interact with the mDia formins [45], is not known.

Finally, Rac itself could act to provide a negative feedback loop to restrict its activity to one region of the plasma membrane via a Rac-WAVE-Arp2/3-Arpin route [46] (Figure 2). Arpin is an inhibitor of the Arp2/3 complex. An alternative loop involves Rac recruiting its own GAP, such as srGAP1 [47].

### Limiting Rho activity during lamellipodium-driven migration

Although RhoA is active at the leading edge of lamellipodia (see above), high levels of RhoA/ROCK activity induce actomyosin-mediated retraction of lamellipodia and inhibit this type of migration [48]. For example, cells switch from lamellipodium-driven migration to bleb/lobopodium-driven migration when RhoA/ROCK activity goes up [49•, 50].

Most pathways so far implicated in neuronal migration in the developing brain appear to converge on regulating RhoA activity [51], probably because high RhoA activity impairs migration. The atypical Rho members Rnd2 and Rnd3 are expressed in different regions and timepoints during cortical development. They both promote migration by suppressing RhoA activity [52]. Rnd proteins are known to activate the Rho-specific p190RhoGAP to reduce RhoA activity [53]. RhoA is also directly phosphorylated by the kinase Mst3, reducing its activity and hence promoting migration of neurons in the cortex [54^•^]. Mst3 in turn is part of the STRIPAK complex, which includes the cerebral cavernous malformation 3 (CCM3) protein [55]. Deletion of CCM3 inhibits migration of cortical neurons, also by increasing RhoA activation [56]. Conversely, the Semaphorin receptor Plexin B2 binds to and titrates down Rnd3, thereby maintaining appropriate levels of active RhoA required for neuronal migration [57].

### Contact inhibition of migration: suppressing lamellipodia

Contact inhibition of migration can occur between two cells moving using lamellipodium-based migration [7]. When two cells meet, the lamellipodia stop extending, are retracted and eventually the cells extend lamellipodia in a different direction [58]. This has been beautifully visualised *in vitro* and *in vivo*. Contact inhibition is important for spacing of *Drosophila* haemocytes (macrophage-like cells) in the developing larva [59]. In several mammalian cell types, contact inhibition is mediated by EphA receptor-induced activation of RhoA/ROCK signalling to induce local retraction of lamellipodia at sites of cell•cell contact [7]. In neural crest cells *in vivo*, the Wnt-PCP (planar cell polarity) pathway activates RhoA and inhibits Rac1 upon cell•cell collision, thereby inhibiting migration [60]. Microtubule catastrophe is also increased in neural crest cells at sites of contact inhibition, mediated by inhibition of the Rac1 GEF Trio [61]. This is consistent with a role for Rac in stabilizing microtubules [62].

## Migration in 3D and *in vivo*: different types of protrusions?

During cancer cell migration in 3D, degradation of extracellular matrix is usually required, and is driven at localized protrusions known as invadopodia [63]. Rho GTPases are well known to contribute to invadopodial protrusions [64]. Cdc42 in particular is involved in formation of invadopodia, acting through its target N-WASP, and several Cdc42 GEFs have been implicated in invadopodia [63]. In 3D environments Rac1, PAK1 and the WAVE complex inhibit invasion and matrix degradation [65, 66••], perhaps in part by inhibiting Cdc42 activity.

Two modes of migration of fibroblasts have been described in 3D: one elongated mode driven by Cdc42 and Rac1, which is lamellipodium-dependent, the other involves ‘lobopodia tm), driven by Rho/ROCK and myosin II [11]. The balance between the two types of migration depends on the elasticity of surrounding matrix: more pliable matrices favour Rho/ROCK-driven migration. Similarly, melanoma cells shift between Rac-mediated lamellipodium-based migration and rounded Rho/ROCK-driven bleb-based migration, depending on the conditions [67]. The Rac/Cdc42 GEF β-PIX has a specific role during fibroblast migration in collagen [49^•^]: β-PIX-depleted fibroblasts have lost polarized Cdc42 but not Rac1 activity, and have hyperactive RhoA. β-PIX interacts with srGAP1, which is normally needed to suppress RhoA activity (although srGAP1 also acts as a GAP for Rac1, see above), and thus the β-PIX/srGAP1 complex mediates Cdc42/RhoA crosstalk.

## Filopodia and cell migration

Filopodia are observed on many cell types and are implicated in directed cell migration and neuronal guidance [68]. Filopodia can also mediate initial cell•cell contact when epithelial cells are moving towards each other [69], and are observed in the leading cells during angiogenesis [70^••^]. Fibroblasts lacking Arp2/3 function predominantly use filopodia for migration [26, 27]. Recently, filopodia have also been implicated in long-range signalling and communication between cells [71]. For example, Cdc42/WASP-driven filopodia transport Wnt8a to responding cells during neural plate formation in zebrafish [72^••^].

Cdc42 is the best characterized Rho GTPase involved in filopodium formation (Figure 1), acting predominantly through formins [20]. Several other Rho GTPases can induce filopodia under different contexts. RhoF induces filopodia through the formins mDia1 and mDia2 [45]. RhoD overexpression induces filopodium-like protrusions, at least in part by interacting with the WASP-related WHAMM protein and/or mDia3C [37, 73]. Multiple proteins in addition to Rho GTPases are important in generating filopodia [68]. These include fascin, which bundles actin filaments in filopodia [74]. Interestingly, the binding of fascin to actin filaments is stimulated by Rho/ROCK signalling, which induces fascin interaction with the ROCK-activated LIMK1/2 [75].

Recent studies indicate that filopodia are important for both lamellipodium-driven and bleb-driven migration *in vivo*. For example, neural crest cells migrate using lamellipodia [1]. In zebrafish, downregulation of fascin led to defective guidance of cranial neural crest cells [76], supporting an important role for filopodia in directed migration. In zebrafish primordial germ cells, which use bleb-driven migration, filopodia were not required for migration itself but for optimal chemotaxis to the chemokine CXCL12 [77^••^]. Interestingly, filopodia appeared to capture CXCL12 and bring it back to the cell body, where a bleb subsequently formed. In addition, filopodia were required for polarized accumulation of active Rac1 at the front of cell, consistent with a model where Cdc42 mediates localized Rac1 activation (see above). However, filopodia appear less important for guidance of angiogenic sprouts in zebrafish, even though the leading cells of sprouts have abundant filopodia [78]. In this system, filopodia were suppressed using low concentrations of latrunculin B, which prevents actin monomers from polymerizing.

## Bleb-driven cell migration

**Figure 3:**
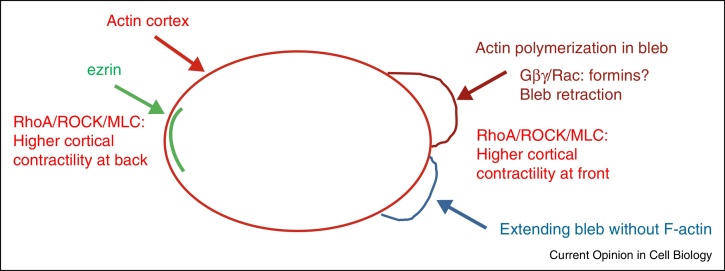
Rho GTPases in bleb-driven migration. The predominant Rho GTPase involved in bleb-driven migration is RhoA, acting through ROCK to stimulate myosin light chain phosphorylation (pMLC) and hence cortical actomyosin contractility, which is higher at the front and back of the cell than on the sides. At the back of the cell, ezrin is associated with the actin cortex and reduces bleb formation [111]. At the front of the cell, actomyosin contractility leads to focal detachment of the plasma membrane from the actin cortex to form blebs, which initially do not contain actin filaments (shown in blue). Subsequently actin polymerizes on the bleb membrane to stabilize the protrusion, eventually leading to bleb retraction. This could be mediated by Rac, which as activated at the front of blebbing primordial germ cells in zebrafish by the G-protein subunits Gβγ.

Bleb-based migration is driven by cortical actomyosin contractility (Figure 3), and is associated with high levels of active RhoA/ROCK signalling [79]. So far, a role for RhoC in bleb-based migration has not really been addressed, but it is relevant that RhoC is frequently upregulated in metastasis, and is associated with metastasis particularly in melanoma [80], which involves predominantly rounded bleb-driven migration [67]. Bleb-based migration is rarely observed in 2D culture conditions, but is frequently observed *in vivo* and in confined environments or on low-adhesion 3D systems *in vitro*. For example, Dictyostelium normally use ‘pseudopod tm)-based migration (equivalent to lamellipodia), but convert to bleb-based chemotaxis under agarose of increasing stiffness and thus higher mechanical resistance, which requires myosin II [81]. Similarly, stable bleb-based migration of isolated zebrafish germ layer progenitor cells in confined environments *in vitro* is driven by Rho/ROCK signalling and actomyosin contractility [82^•^]. These cells have high speed and persistence. This involves lysophosphatidic acid (LPA), which is well known to activate RhoA/ROCK through LPA receptors [83]. Indeed, localized LPA delivery to germ layer progenitor cells induces localized myosin II accumulation, at what then becomes the back of the cell [82^•^]. This resembles the uropod at the back of migrating leukocytes, which is similarly enriched in myosin II [84].

Cells can transition rapidly between bleb-based and lamellipodium-based migration *in vivo*, which may reflect their adaptation to variations in extracellular matrix pliability and structure. For example, during early zebrafish development, individual involuting mesodermal cells extend blebs interchangeably with lamellipodia over time [85]. Primordial zebrafish germ cells use predominantly bleb-based migration to migrate towards CXCL12, but Rac1 is also active at the leading edge (Figure 3) [86]. Higher pH is required at the front of these cells during migration both for maintaining cell contractility and polarized Rac1 distribution [87^•^]. As with lamellipodium-driven migration, it is important for cells using bleb-driven migration to maintain the correct level of adhesion: germ cells use E-cadherin to gain traction on neighbouring cells as they migrate between them [86].

Under some conditions, cells appear to stabilize bleb-driven migration at the transcriptional level. For example, a LIF/JAK/STAT-driven positive feedback loop acts to maintain Rho/ROCK activity in melanoma cells [88]. Indeed, melanoma cells with high actomyosin contractility and rounded morphology secrete many factors, including MMPs, which promote bleb-driven migration through a positive feedback loop [89]. Recently Rac1 acting in the nucleus has been found to regulate nuclear morphology and promote actomyosin contractility and invasion [90^•^], although whether Rac1 is regulating transcription in the nucleus in this case is not clear.

## Collective cell migration

Many cell populations migrate collectively during development, including epithelial cells, endothelial cells and neural crest cells [91]. Collective cell migration is usually driven by lamellipodia and filopodia in the leading cell, and suppression of these protrusions in the other cells. For example, in Drosophila border cell migration, Rac1 is required to be active selectively in the leading cell, and is suppressed in other cells of the cluster via E-cadherin-mediated adhesion between the leader and followers [92^••^]. Localized photoactivation of Rac1 in one cell leads to extension of a protrusion, which guides the migration of the border cell cluster [93]. Neural crest cells are mesenchymal but migrate coordinately during development, so have aspects of collective migration behaviour [94]. In migrating neural crest cells, Rac1 activity is required at the leading edge. RhoU contributes to migration of cranial neural crest cells by acting together with Rac1 and PAK [95].

Rac1 and Cdc42 are also important in the leading endothelial cells during angiogenic sprouting. In zebrafish, Cdc42 is activated by ARHGEF9, which then activates the formin FMNL3 to induce filopodia during angiogenic sprouting of the caudal vein plexus [70^••^]. Rac1 is required for sprouting of endothelial cells *in vitro*, and actomyosin contractility suppresses sprouting [96]. However, *in vivo* Rac1 only appears to contribute to angiogenesis in the absence of the integrin αvβ3 [17]. The ELMO/DOCK180 RacGEF acts via Rac1 and PAK to protect endothelial cells from apoptosis and hence promotes formation of blood vessels indirectly [97].

It is important to maintain stability of adherens junctions between collectively migrating cells, which in turn signal to keep Rac1 active at the front of the leading cells. For example, in migrating neural crest cells, the adherens junction protein N-cadherin suppresses Rac1 activity at cell•cell junctions, whereas Rac1 is active in the leading edge [98]. During collective endothelial migration, the extracellular signalling molecule Ang-1 promotes adherens junction stability via aPKCζ and the adherens junction protein β-catenin, leading to selective activity of Rac1 at the leading edge [99]. As well as regulating Rac1 activity, adherens junctions are important for determining directionality of collectively migrating astrocytes by ensuring Cdc42 is recruited to the front of leader cells [100], where it presumably leads to Rac1 activation.

The role of RhoA in adherens junction signalling is more controversial. On the one hand, Rnd3 contributes to collective cell migration in epithelial cancer cells by repressing ROCK activity and keeping actomyosin contractility at cell•cell junctions low [101]. RhoJ is required for tumour angiogenesis in mice, and acts by reducing Rho/ROCK activity [102^••^]. Conversely, in angiogenesis in mice, a Raf1/ROCK2 complex activates actomyosin contractility selectively at adherens junctions to mediate the maturation of adherens junctions essential for collective migration [103]. It is likely that Rho/ROCK activity is initially required to form adherens junctions through contraction of actin filaments parallel to the junctions, but subsequently needs to be reduced to stabilize the contacts.

## Migration across other cells

Cells *in vivo* frequently migrate between other cells. For example, during development primordial germ cells migrate between multiple cell types to reach the sites where gonads will form [104]. Leukocytes constantly migrate across endothelial cells and epithelial cells to enter and exit tissues [105, 106]. Cancer cells also migrate in and out of blood vessels during metastasis [107]. Rho GTPases contribute to transmigration in both cell types. Endothelial Rac1 and RhoG promote the initial interaction between leukocytes and endothelial cells through adhesion receptor clustering [108]. On the other hand, strengthening endothelial cell•cell junctions by inhibiting PI3Kα and Rac1 reduces transendothelial migration of leukocytes [108, 109]. These two distinct roles of Rac1 demonstrate the importance of timing and localization of Rho GTPase activation in regulating migratory processes. Once they have adhered to endothelial cells, leukocytes extend small protrusions between endothelial cells or through endothelial cells, which then form lamellipodia and filopodia under the endothelial cells. T-cell RhoA is particularly important for transendothelial migration, probably because it is active at both the front and back of transmigrating T-cells [24]. In addition, during T-cell receptor-driven transmigration of T-cells, the GEF Vav and its downstream target Rac contribute to transendothelial migration [110].

## Conclusions and future directions

Cells are remarkably flexible in the ways they migrate, adapting rapidly to changing cues in their environment to extend different types of protrusions and change shape. In some cases they also use transcriptional reprogramming to maintain their ability to move using lamellipodium-driven or bleb-driven migration. Transcriptional changes could be relevant *in vivo* for relatively slowly migrating cancer cells at the edge of tumours, but not during rapid shape changes such as those that occur during leukocyte transendothelial migration. The ability to follow localized Rho GTPase activation in real time *in vivo* will increase our understanding of how these dynamic changes in migration are regulated. So far, most studies on migration have focussed on Rho, Rac and Cdc42 proteins, and we know relatively little about how atypical Rho family members contribute to migration *in vivo*. Animal models investigating how these proteins signal *in vivo* will help resolve their roles in migration.

## References and recommended reading

Papers of particular interest, published within the period of review, have been highlighted as:

• of special interest
•• of outstanding interest

## Figures and Tables

**Table 1:** Rho GTPase family The 20 human Rho GTPases are listed in subfamilies. Reported splice variants and C-terminal lipid modifications are shown. GG, geranylgeranylation; F, farnesylation; P, palmitoylation.

Rho GTPase	Subfamily	Splice variants	C-terminal modifications
RhoA	Rho		GG
RhoB	Rho		GG, F
RhoC	Rho		GG
Rac1	Rac	Extra exon 3b	GG, P
Rac2	Rac		GG
Rac3	Rac		GG
RhoG	Rac		GG
Cdc42	Cdc42	Alternative C-terminal exon	GG, P
RhoJ	Cdc42		GG
RhoQ	Cdc42		GG
RhoU	RhoU/V		P
RhoV	RhoU/V		P
RhoD	RhoD/F		GG
RhoF	RhoD/F		GG
Rnd1	Rnd		F
Rnd2	Rnd		F
Rnd3	Rnd		F
RhoH	RhoH		F
RhoBTB1	RhoBTB		None
RhoBTB2	RhoBTB		None
